# Expression of long non-coding RNA ***MFI2-AS1*** is a strong predictor of recurrence in sporadic localized clear-cell renal cell carcinoma

**DOI:** 10.1038/s41598-017-08363-6

**Published:** 2017-08-17

**Authors:** Ronan Flippot, Roger Mouawad, Jean-Philippe Spano, Morgan Rouprêt, Eva Compérat, Marc-Olivier Bitker, Jérôme Parra, Christophe Vaessen, Frederick Allanic, Quentin Manach, Nizar M. Tannir, David Khayat, Xiaoping Su, Gabriel G. Malouf

**Affiliations:** 1 Department of Medical Oncology, Pitié-Salpêtrière Hospital, Paris, France; 2 University Pierre and Marie Curie, Institut Universitaire de Cancérologie, Paris, France; 3 University Pierre and Marie Curie, INSERM UMRS 1136 Paris, France; 4 Department of Urology, GRC5, Pitié-Salpêtrière Hospital, 75013 Paris, France; 5 Department of Pathology, Tenon Hospital, HUEP, Paris, France; 6 Department of Genitourinary Medical Oncology, The University of Texas MD Anderson Cancer Center, Houston, Texas USA; 7 Department of Bioinformatics and Computational Biology, The University of Texas MD Anderson Cancer Center, Houston, Texas USA

## Abstract

Prediction of recurrence is a challenge for the development of adjuvant treatments in clear-cell renal cell carcinoma (ccRCC). In these tumors, expression of long non-coding RNAs (lncRNAs) are deregulated and closely associated with prognosis. Thus, we aimed to predict ccRCC recurrence risk using lncRNA expression. We identified prognostic lncRNAs in a training set of 351 localized ccRCCs from The Cancer Genome Atlas and validated lncRNA-based recurrence classification in an independent cohort of 167 localized ccRCCs. We identified lncRNA *MFI2-AS1* as best candidate in the training set. In the validation cohort, *MFI2-AS1* expression was independently associated with shorter disease-free survival (Hazard Ratio (HR) for relapse 3.5, p = 0.0001). Combined with Leibovich classification, *MFI2-AS1* status improved prediction of recurrence (C-index 0.70) compared to *MFI2-AS1* alone (0.67) and Leibovich classification alone (0.66). In patients with aggressive tumors (Leibovich ≥5), *MFI2-AS1* expression was associated with dramatically increased risk of relapse (HR 12.16, p < 0.0001) compared to patients with undetectable *MFI2-AS1* who had favorable outcomes. Compared to normal samples, *MFI2-AS1* was upregulated in tumor tissue, and higher expression was associated with metastatic dissemination. Overall, *MFI2-AS1* status improves patient stratification in localized ccRCC, which supports further integration of lncRNAs in molecular cancer classifications.

## Introduction

Almost half of the 300,000 people diagnosed with kidney cancer each year die from cancer progression^1^. Clear cell renal cell carcinoma (ccRCC), more than 75% of renal cell carcinomas^2^, is diagnosed as localized disease in 70% of cases. Partial or radical nephrectomy is standard therapy for stage I–III ccRCC, one third of which will recur.

Improving prognostic classifications is a major challenge. Indeed, adjuvant clinical trials use clinicopathological criteria associated with poor prognosis, such as elevated T stage, high pathological grade or nodal involvement^3–5^. Thus, most patients in these trials belong to intermediate or high-risk groups according to the historical Leibovich classification^6^. However, 70% of patients in the intermediate group and 30% of patients in the high-risk group will not have ccRCC recurrence at 5 years. Thus, many adjuvant trials report negative results for improved recurrence-free survival, and many patients are treated needlessly. New insights in tumor biology are needed to improve prognostic classifications.

Historically, ccRCC is known for alterations of the VHL protein, which leads to the activation of the hypoxia inducible factor pathway that promotes angiogenesis^7^. Recent studies have found alterations of chromatin remodeling genes thought to be crucial to carcinogenesis, such as *BAP1*, *PBRM1* and *SETD2*, in up to 40% of ccRCCs^8^, which might indicate that epigenetic regulation is important for ccRCC progression.

Long non-coding RNAs (lncRNAs), ribonucleic acids with more than 200 bases that act as genome-wide epigenetic regulators, are new players that might be involved in ccRCC ontogeny. Indeed, multiple lncRNAs are reported to be deregulated in ccRCC^9, 10^. Importantly, study of lncRNA signatures might help discriminate malignant and benign tumors^11^ and improve cancer subtype classifications^12^. We recently attempted to study the expression profile of lncRNAs in ccRCC through unsupervised clustering and found 4 different clusters, including a subgroup C2 that was associated with dismal prognosis and aggressive tumor features^13^. Thus, lncRNAs appear to be putative potent predictors of survival in ccRCC.

Despite attempts to classify recurrence risk according to molecular features of ccRCC^14^, strong markers of recurrence easily implemented in clinic are lacking. Thus, we sought to identify lncRNAs strongly associated with prognosis and improve patient stratification with a recurrence risk assessment based on lncRNA expression in patients with localized ccRCC.

## Material and Methods

### Selection of the reference recurrence model

Two scores are validated to evaluate the risk of relapse in localized ccRCC after radical nephrectomy, the UCLA staging system (UISS) and the Leibovich score. The UISS classification includes T and N status, pathological grade and clinical performance status, while the Leibovich score includes T and N status, tumor size, pathological grade and necrosis.

Several considerations made us choose the Leibovich score over the UISS classification. Published literature suggests that the Leibovich scoring system is more accurate than the UISS classification regarding the prediction of outcomes in localized ccRCC after radical nephrectomy^15^. In addition, the Leibovich score was the standard chosen for the recent evaluation of molecular classifications for recurrence in localized ccRCC^14^. Finally, the recent major adjuvant clinical trials in clear-cell renal cell carcinoma (ccRCC) selected patients based on modified UISS risk groups that were not validated^16–18^. These considerations made the choice of the Leibovich score the most relevant validated classification for the evaluation of recurrence risk in localized ccRCC.

Leibovich classification stratifies patients with low (0–2), intermediate (3–5) or high (>5) recurrence risk. Current adjuvant trials select patients on the basis of aggressive pathological features that belong to both intermediate and high-risk groups^17^. Thus, we chose to discriminate between patients with Leibovich scores <5 and those with scores ≥5 to account for intermediate-risk patients with aggressive features and comply with current adjuvant trials.

### Patients

We evaluated two independent retrospective cohorts: a discovery set from the TCGA database^19^ and a validation set from Pitié-Salpêtrière Hospital.

The discovery set included patients with stages I, II and III ccRCC who underwent partial or radical nephrectomy between August 2005 and January 2016, with available RNA-sequencing data and clinical annotations. Clinical data included disease-free survival (DFS), overall survival (OS) and follow-up (FU).

The validation cohort included patients with available fresh frozen tumor samples from stages I, II and III ccRCC. The samples underwent central review by an expert pathologist according to the guidelines of the International Society of Urological Pathology^20^. Patients with TFE3 or TFEB translocations and hereditary cancers were excluded because the natural history and oncogenic alterations might differ from those associated with sporadic ccRCC. Other exclusion criteria were insufficient RNA yield (<2,000 ng) and FU less than 3 months. Clinicopathological data included age, gender, tumor size according to TNM staging, pathological grade, sarcomatoid or rhabdoid features, necrosis, renal vein thrombosis, Leibovich classification, tumor recurrence (local or distant) and DFS. Up to 4 samples from the primary tumor were collected in six patients to assess tumor heterogeneity for lncRNA expression. For exploratory analyses, matched normal tissue samples from 21 patients were included, as well as additional primary tumors from 21 patients who had metastases at diagnosis. Each tissue sample was collected through surgical resection.

All patients had previously provided written informed consent for tumor collection and analysis. The study was approved by the ethical committee of Pitié-Salpêtrière Hospital (IDF-6, Ile de France). The collection and use of tissues followed ethical procedures formulated in the Helsinki Declaration.

### Procedures

In the discovery set, RNA expression had been assessed by RNA sequencing from fresh frozen primary tumor samples. Total RNA from each sample was converted into cDNA with the Illumina TruSeq RNA sample preparation kit, and cDNA was sequenced with the Illumina HiSeq. 2000 platform according to the manufacturer’s instructions.

For the validation set, expression of target lncRNA MFI2-AS1 was assessed with real-time quantitative PCR from fresh frozen primary tumor samples. Total RNA was extracted from tumor samples using the ThermoFisher Scientific PureLink® RNA mini kit. Total RNA quantitation was performed with ThermoFisher Scientific Multiskan GO® microplate spectrophotometer. Supplementary extractions were performed if the total RNA yield was less than 2000ng of total RNA. After extraction, total RNA was converted into cDNA using the ThermoFisher Scientific high capacity RNA to cDNA kit. Real-time quantitative PCR was performed using the ThermoFisher Scientific TaqMan® master mix and predesigned primer and probe sets for *MFI2-AS1* (reference: Hs04274310_g1). Each experiment was conducted in duplicate, with 40ng of RNA in each well. The experiment was performed on a Roche LightCycler® 480 platform up to 40 PCR cycles, according to the manufacturer’s instructions. Relative quantification of gene expression was performed using the Delta Cp method^21^. Detectable expression was defined by a crossing point (Cp) <40, with similar expression in the duplicate experiment (differential Cp <1) and adequate curve aspect (Supplementary Figure 1). We chose *PPIA* as the reference gene for relative quantitation of gene expression, as it has been reported as a top-ranked reference gene for gene expression analysis in ccRCC^22–24^. To validate this approach, we internally evaluated multiple reference genes in addition to *PPIA*, including *GUSB*, reported as a stable marker in ccRCC^25^, as well as *18 S* and *GAPDH*. We assessed the expression of a known target lncRNA, *HOTAIRM1*, in five reference ccRCC samples, using RNA-sequencing and qRT-PCR with each reference gene. PPIA was the most accurate reference gene, allowing the highest correlation between qRT-PCR and RNA-sequencing data (Spearman r = 0,800).

### Endpoints and statistical analysis

The primary endpoint was the impact of lncRNA expression to predict disease recurrence in localized ccRCC. The primary outcome for both cohorts was DFS, defined as the time from initial surgery to first relapse, identified by physical examination, biopsy or imaging. DFS was censored at the last FU or death in patients without documented recurrence.

The secondary endpoint in the training set was to identify associations between *MFI2-AS1* expression and genome-wide expression changes. In the validation cohort, secondary endpoints were DFS according to clinicopathological subgroups, DFS according to lncRNA expression and Leibovich classification, and differential lncRNA expression between normal tissue, localized tumors, and metastatic tumors in the validation cohort.

To identify candidate prognostic lncRNAs, we studied 1934 lncRNAs previously identified^13^ as significantly expressed in the TCGA ccRCC cohort, with reads per kilobase per million mapped reads (RPKM) ≥1 in at least 10% of ccRCC samples. The choice of candidate lncRNAs was based on their association with DFS and OS, expression relative to tumor stage, oncogenic potential, and availability for standard gene expression assessment. A multiple Cox regression model was used to identify lncRNAs associated with DFS (false discovery rate (FDR) <0.01) and OS (FDR <0.05). T-tests were used to compare lncRNA expression between primary tumors from metastatic patients, localized tumors, and normal tissue.

We sought to identify pathways and mechanisms that could be altered by the dysregulation of *MFI2-AS1* expression. In TCGA dataset, we studied differential gene expression in the top 10% of tumors expressing *MFI2-AS1* compared to the bottom 10%. We defined upregulated genes by a twofold increased expression FDR < 0.05 and p < 0.05. We performed functional annotations of upregulated genes with Gene Ontology (GO) terms using DAVID functional annotation tool.

In the validation set, the correlation between lncRNA expression and DFS was estimated by the Kaplan-Meier model. Comparison of survival curves in univariate analysis was performed with the log-rank test. A multivariate Cox regression model was used in the analysis. In the survival models, the p-value was calculated under the null hypothesis of a hazard ratio (HR) of 1. C-statistics were determined to assess model adequacy and were compared to the Leibovich classification accuracy. Associations between lncRNA expression and clinicopathological data were evaluated with the Fisher exact test or Wilcoxon test, depending on the variables.

### Data Availability

The datasets generated during and/or analysed during the current study are available from the corresponding author on reasonable request.

## Results

### LncRNA *MFI2-AS1* as top-rank marker of ccRCC recurrence

**Figure 1:**
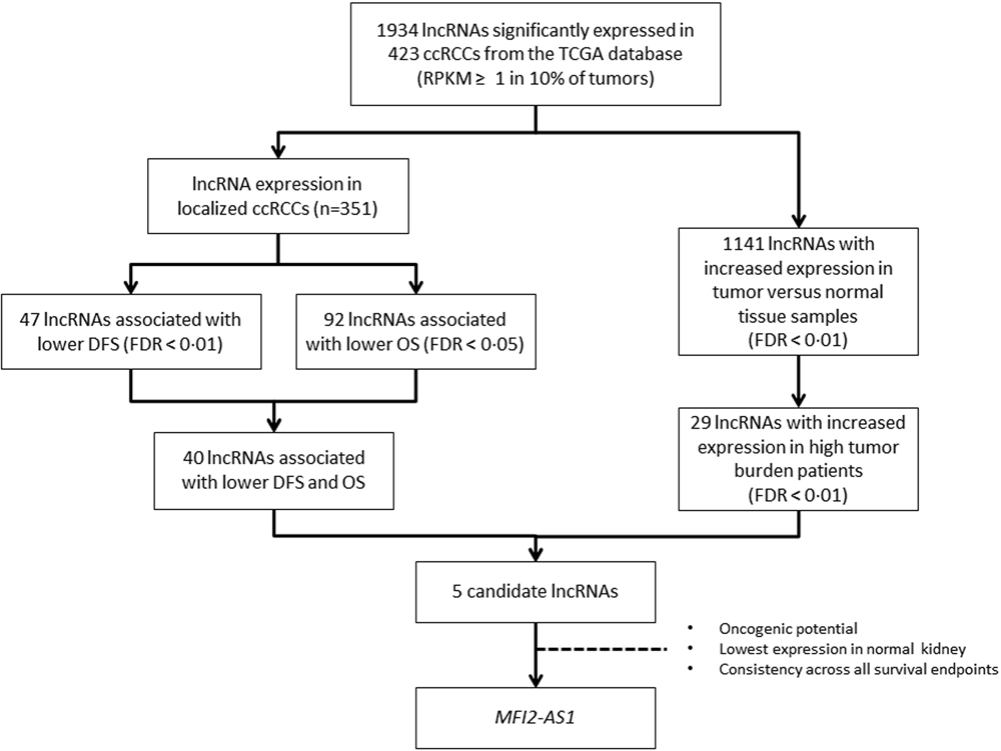
Selection of prognostic long non-coding RNA in the discovery set from The Cancer Genome Atlas.

We analyzed 1934 lncRNAs expressed in tumors from the TCGA ccRCC cohort^19^ with appropriate annotations and RNA-sequencing data (N = 423). In 351 patients with localized disease (Table 1), we looked for lncRNAs associated with shorter DFS and identified 47 statistically significant lncRNAs (adjusted p-value < 0.01). Of those, 40 lncRNAs were also associated with shorter OS (adjusted p-value < 0.05) (Fig. 1 and Supplementary dataset 1).

**Table 1:** Clinicopathologic characteristics of the patients in the training and validation sets.

Variable		Training set population with localized ccRCC		Validation set population with localized ccRCC	
Population		N = 351	%	N = 167	%
Gender	*Male*	224	64	111	66
	*Female*	127	36	56	34
T stage	*1*/*2*	244	70	144	86
	*3*/*4*	107	30	23	14
Grade	*1*/*2*	173	49	110	66
	*3*/*4*	178	51	57	34
Sarcomatoid	*Yes*	—	—	14	8
	*No*	—	—	153	92
Rhabdoid	*Yes*	—	—	6	4
	*No*	—	—	161	96
Necrosis	*Yes*	—	—	12	7
	*No*	—	—	155	93
Thrombosis	*Yes*	—	—	7	4
	*No*	—	—	160	96
Leibovich score	*Low*	—	—	107	64
	*Intermediate*	—	—	40	24
	*High*	—	—	20	12
Leibovich score	<*5*	—	—	135	81
	≥*5*	—	—	32	19
Age (median, years)		61 (26–90)		63 (31–90)	
Follow-up (median, months)		38 (0–122)		41 (3–122)

To determine which lncRNAs might be oncogenic and important in ccRCC metastasis, we focused on lncRNAs overexpressed in cancer versus normal kidney tissue (1134 lncRNAs, p < 0.01) or overexpressed in metastatic kidney cancer compared with non-metastatic kidney cancer (71 lncRNAs, p < 0.01). We found 5 candidate lncRNAs: *MFI2-AS1*, *SNHG15*, *RP4-584D14*.*7*, *RP11-465L10*.*7*, and *CTC-444N24*.*8*. Of those, only *MFI2-AS1* ^26^ and *SNHG15* ^27, 28^ have been reported as putative oncogenes in other cancers. *MFI2-AS1* had the lowest expression in normal kidney tissue and was thus chosen as the top candidate (Fig. 1 and Supplementary dataset 1).

We investigated the implication of *MFI2-AS1* in regard to the lncRNA-based unsupervised clustering of ccRCC^13^. *MFI2-AS1* was overexpressed in cluster 2 as compared to other clusters (Fold change = 3.02, p-value = 8.52 × 10^−15^, FDR = 5,15 × 10^−13^), which was associated with dismal prognosis and aggressive tumor features. Correlations between *MFI-2AS1* expression and copy-number variations found a high correlation with amplification of its locus at 3q29 (correlation coefficient = 0.34, corrected p-value = 4,44 × 10^−16^).

**Figure 2:**
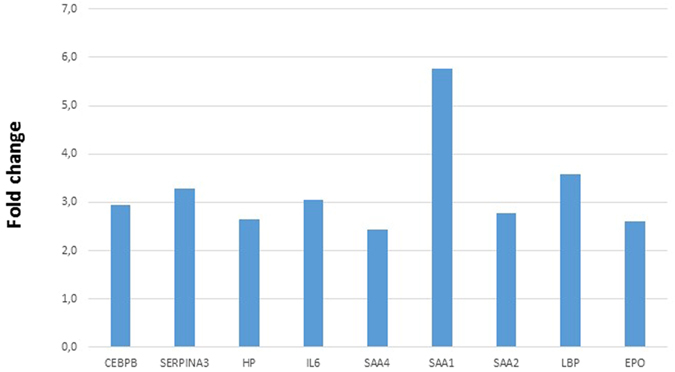
Differential expression of genes involved in acute-phase response in the top 10% of tumors expressing *MFI2-AS1* compared to the bottom 10%. *CEBPB*: CCAAT/enhancer binding protein beta(CEBPB), *EPO:* erythropoietin, *HP:* haptoglobin, *IL6*: Interleukin 6, *LBP:* lipopolysaccharide binding protein, *SERPINA3*: Serpin A3, *SAA:* serum amyloid protein.

Among 13656 genes, we identified 541 genes that were upregulated in tumors with high expression of *MFI2-AS1*. Functional analysis revealed that these genes were associated with acute phase inflammatory response (Fig. 2 and Supplementary dataset 2). These genes include haptoglobin, erythropoietin, interleukin 6, *CEBPB*, lipopolysaccharide binding protein, Serpin A3, and serum amyloid proteins A1, A2, A4.

### *MFI2-AS1* is a potent and independent predictive marker for ccRCC recurrence

The validation cohort included 204 patients with localized ccRCC. Five patients were excluded for insufficient RNA yield, another five for insufficient quality of PCR data (attributed to degraded RNA), and 27 were excluded for insufficient FU. Characteristics of the analysis population are reported in Table 1. The median FU was 41 months (range, 3–122 months); median age at diagnosis was 63 years (range, 31–90 years), with a male to female ratio of 1.9 to 1. Most patients had low T stage (86% of T stage 1–2) and low-grade tumors (66% with grade 1–2). Less than 10% of patients had sarcomatoid or rhabdoid components, tumor necrosis or renal vein thrombosis in pathological review. According to Leibovich scores, 107 (64%) patients had low recurrence risk, while 40 (24%) and 20 (12%) had intermediate or high risk, respectively. Overall, 135 (81%) patients had Leibovich scores <5 and 32 (19%) patients had Leibovich scores ≥5.

Analysis of *MFI2-AS1* expression by quantitative PCR revealed that two thirds of tumors (66.5%) did not express *MFI2-AS1* at a detectable level. We thus plotted a receiver operating characteristic (ROC) curve for recurrence based on the relative expression of *MFI2-AS1* in the validation cohort. Analysis of the ROC curves determined that the best performing stratification of patients to assess recurrence risk was based on the discrimination between the presence or absence of detectable *MFI2-AS1* transcripts (Supplementary Figure 2).

**Figure 3:**
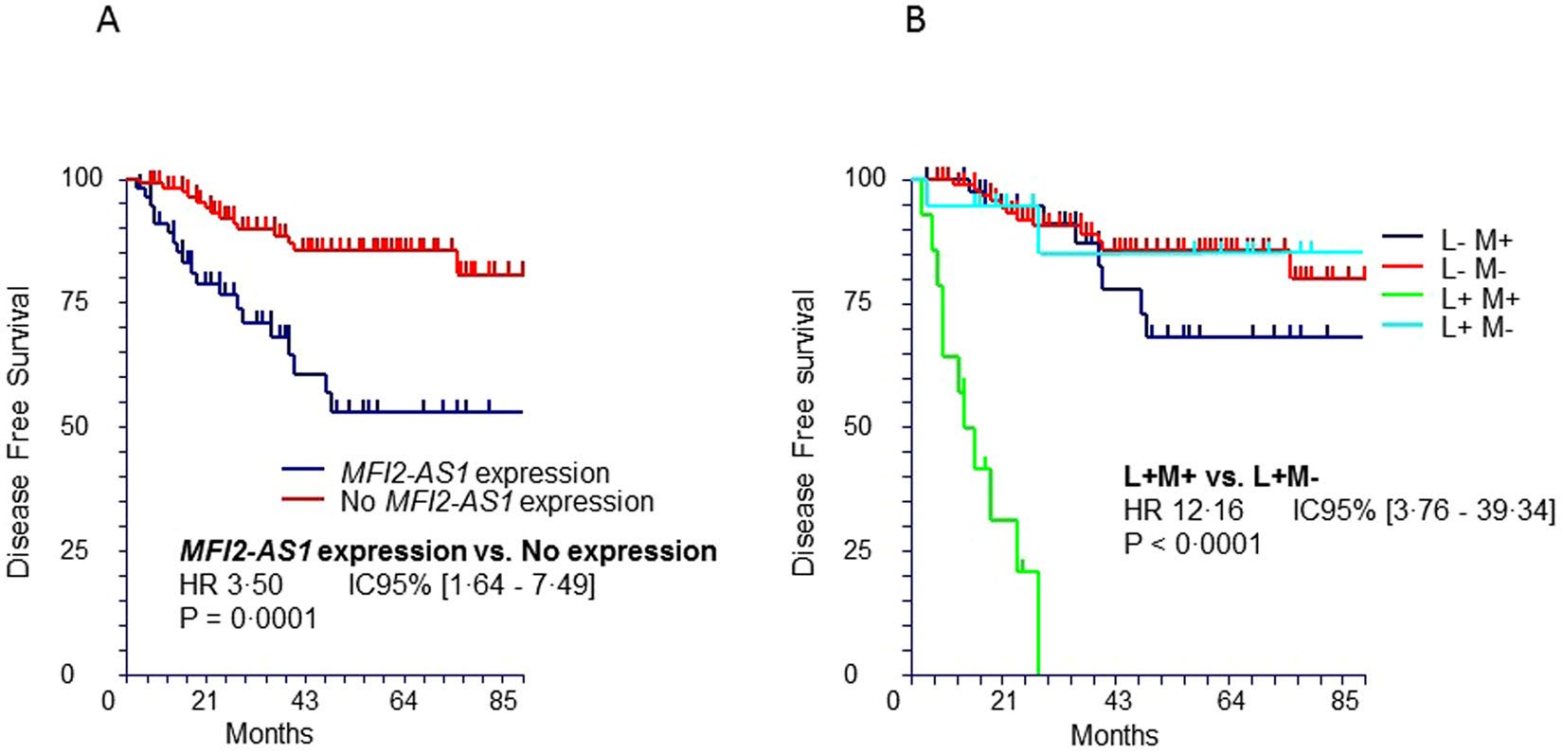
Kaplan-Meier estimates for disease-free survival (DFS) in the validation set. (**A**) DFS according to *MFI2-AS1* expression in the entire cohort. (**B**) DFS according to *MFI2-AS1* expression and Leibovich subgroups. L+ M+ (N = 14): Leibovich ≥5+ MFI2-AS1 expression. L+ M− (N = 18): Leibovich ≥5+ No MFI2-AS1 expression. L− M+ (N = 42): Leibovich <5+ MFI2-AS1 expression. L− M− (N = 93): Leibovich <5+ No MFI2-AS1 expression.

*MFI2-AS1* expression was associated with significantly shorter DFS in the univariate analysis, with a HR for relapse of 3.5 (95% CI [1.64–7.49], p = 0.0001) (Table 2 and Fig. 3A). Of note, the median DFS was not reached in both groups. Patients with *MFI2-AS1* expression also had more aggressive tumor features, including sarcomatoid or rhabdoid contingents (p = 0.0424), tumor necrosis (p = 0.0219) or renal vein thrombosis (p = 0.0429) (Table 3). While patients with MFI2-AS1 expression more often had high Leibovich scores (p = 0.0205), this association was not reported when comparing Leibovich scores ≥5 to those <5. No association was found between *MFI2-AS1* and tumor grade (p = 0.1078). Other variables associated with increased DFS in univariate analysis were renal vein thrombosis (p < 0.0001), necrosis (p = 0.0008), grade 3–4 (p = 0.0003), sarcomatoid or rhabdoid contingent (p < 0.0001), and Leibovich score ≥5 (p < 0.0001) (Table 2).

**Table 2:** Hazard ratio for disease-free survival according to patient and tumor characteristics.

Variable	Hazard ratio [95% CI]	p-value in univariate analysis
**Age** (years) >65 vs <65	1.52 [0.77–3.03]	0.2238
**Gender** Male/Female	1.14 [0.55–2.36]	0.7267
**Thrombosis** Yes/No	7.57 [0.58–99.38]	<0.0001
**Necrosis** Yes/No	4.02 [0.87–18.60]	0.0008
**Sarcomatoid or rhabdoid** Yes/No	5.50 [1.49–20.40]	<0.0001
**Tumor grade** 3–4/1–2	3.27 [1.55–6.93]	0.0003
**T Stage** 3–4/1–2	2.11 [0.72–6.19]	0.0721
**Leibovich score** ≥5/<5	4.11 [1.52–11.10]	<0.0001
**Leibovich score** *High vs*. *intermediate*	3.33 [1.11–9.99]	<0.0001
*Intermediate vs*. *low*	1.99 [0.80–4.93]	
*High vs*. *low*	7.29 [1.63–32.58]	
**MFI2-AS1 expression** Yes/No	3.50 [1.64–7.49]	0.0001
	**Hazard ratio [95% CI]**	**p-value in multivariate analysis**
**Tumor grade** *3–4/1–2	3.37 [1.68–6.76]	0.0006
**MFI2-AS1 expression** *Yes/No	3.62 [1.80–7.27]	0.0003
**Leibovich score** ** ≥5/<5	5.18 [2.49–10.81]	<0.0001
**MFI2-AS1 expression** **Yes/No	4.24 [2.07–8.70]	0.0001

*Multivariate analysis according to MFI2-AS1 expression, T stage and grade. **Multivariate analysis according to MFI2-AS1 expression and Leibovich score.

**Table 3:** Characteristics of the patients according to MFI2-AS1 expression.

Variable		Validation set		
Population		MFI2-AS1 expression N = 56	No MFI2-AS1 expression N = 111	p-value
Age (years)	>63	31	57	0.7429
	<63	25	54	
Gender	*Male*	37	74	1.000
	*Female*	19	37	
T Stage	*3*/*4*	9	14	0.6352
	*1*/*2*	47	97	
Tumor grade	*3*/*4*	24	33	0.1078
	*1*/*2*	32	78	
Sarcomatoid or rhabdoid	*Yes*	11	9	0.0424
	*No*	45	102	
Necrosis	*Yes*	8	4	0.0219
	*No*	48	107	
Thrombosis	*Yes*	5	2	0.0429
	*No*	51	109	
Leibovich	≥*5*	13	18	0.2961
	<*5*	43	93	
Leibovich	*Low*	30	77	0.0205
	*Intermediate*	14	26	
	*High*	12	8	

The prognostic impact of *MFI2-AS1* expression remained highly significant in the multivariate analyses (Table 2). As the Leibovich score involves tumor grade, we used two distinct Cox regression models to avoid statistical bias. The first analysis included *MFI2-AS1* expression and Leibovich score (<5 or ≥5), which remained independent from each other, with a p-value of 0.0001 for *MFI2-AS1* and <0.0001 for the Leibovich score. The second analysis included *MFI2-AS1* expression and tumor grade, which remained independent, with respective p-values of 0.0003 and 0.0006.

### Intratumor variation of *MFI2-AS1* expression

Spatial and temporal heterogeneity of clear-cell renal cell carcinoma have been recently uncovered^29–31^, and contribute to challenging the reproducibility of biomarker studies. Herein we studied intratumor heterogeneity of *MFI2-AS1* expression in 17 samples from 6 primary tumors, with 2 to 4 distinct regions collected from each tumor.

All tumors had documented *MFI2-AS1* expression. Multiregion analysis for *MFI2-AS1* expression revealed that only one tumor had differential detection of *MFI2-AS1* with absence of *MFI2-AS1* expression reported in one region out of three studied (Supplementary Table 1). Normalized quantitation of *MFI2-AS1* expression highlighted high variation between tumor samples. Two tumors had very similar *MFI2-AS1* expression levels across the collected samples, with variations under 1,8 fold, while the three other tumors had variations from 7 to 91-fold.

### Improved stratification of ccRCC recurrence risk using *MFI2-AS1* expression and Leibovich score

To evaluate the clinical utility of *MFI2-AS1* expression for DFS prediction, we stratified Leibovich subgroups according to *MFI2-AS1* expression, namely Leibovich ≥5+ *MFI2-AS1* expression (L+ M+), Leibovich ≥5+ no expression of *MFI2-AS1* (L+ M−), Leibovich <5+ *MFI2-AS1* expression (L− M+), and Leibovich <5+ no expression of *MFI2-AS1* (L− M−). The L+ M− group had a spectacular improvement in DFS compared to the L+ M+ group. Indeed, in the L+ M+ group, median DFS was 10 months, which was not reached in the L+ M− group. The HR for recurrence in the L+ M+ group compared to the L+ M− group was 12, 95% CI [3.76–39.34], p < 0.0001 (Fig. 3B). Stratification of Leibovich groups according to *MFI2-AS1* expression reclassified 56% of the patients with Leibovich scores ≥5 (18/32 patients), who were ultimately considered to have favourable outcomes.

The recurrence risk did not differ between the L− M+ and L− M− groups according to *MFI2-AS1* expression. There was also no significant difference in DFS and recurrence risk between the L+ M− group and both the L− M+ and L− M− groups.

Overall, the classification combining the Leibovich score and *MFI2-AS1* expression more accurately estimated the risk of ccRCC recurrence than each characteristic alone, with a C-statistic of 0.70 compared to 0.64 for *MFI2-AS1* stratification alone and 0.66 for Leibovich stratification alone (Supplementary Figure 2).

The results observed in these survival analyses were reproduced with different cutoffs for the Leibovich score, which ultimately led to similar significant results (Supplementary Figure 3).

### Increased *MFI2-AS1* expression is associated with metastatic disease

**Figure 4:**
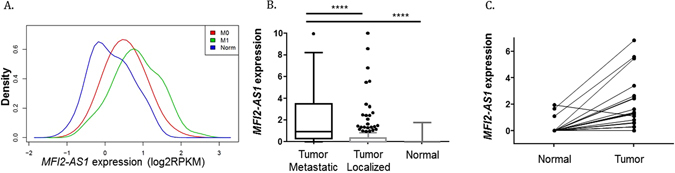
Comparison of *MFI2-AS1* expression in normal tissue, localized tumors, and primary tumors from metastatic patients. (**A**) Development cohort. M0: localized tumors, M1: primary tumors from metastatic patients, Norm: normal tissue. (**B**) Validation cohort. (**C**) Expression of *MFI2-AS1* in the validation cohort between localized ccRCCs and matched normal tissue.

In the training set from TCGA, *MFI2-AS1* expression increased in primary tumors from metastatic patients (N = 72) compared to localized tumors (N = 351), and in localized tumors compared to normal tissue from the entire cohort (N = 423) (Fig. 4A). Similar results were observed in the validation set between primary tumors from metastatic patients (N = 21), localized tumors (N = 167), and normal tissue (N = 21) (Fig. 4B). In paired normal tissue and localized tumor samples, we found a significant increase in *MFI2-AS1* expression (p < 0.0001). Notably, out of 21 matched localized tumor and normal tissue samples, only 3 patients had detectable expression of *MFI2-AS1* in normal kidney tissue, while all 21 patients had expression of *MFI2-AS1* in the primary tumor (Fig. 4C). Together, these data confirm that *MFI2-AS1* is upregulated in tumor samples compared to normal tissue and that higher expression of *MFI2-AS1* is associated with metastatic dissemination.

## Discussion

Our work supports the use of lncRNA *MFI2-AS1* as a potent and independent biomarker to predict disease recurrence in localized ccRCC. Patients with M*FI2-AS1* expression are nearly four times more likely to experience disease relapse after partial or radical nephrectomy. More importantly, the implementation of *MFI2-AS1* status along with Leibovich score stratifies patients who have tumor features usually associated with aggressive disease into two populations with drastically different outcomes. Given the development of adjuvant trials involving immune checkpoint inhibitors and antiangiogenics in localized ccRCC^32, 33^, identifying patients with high recurrence risk is crucial to increase the likelihood of positive outcomes and ensure personalized care.

Indeed, multiple trials have failed to demonstrate improved DFS in the adjuvant setting^16, 34^. The only trial that met its primary endpoint in this setting was based on a careful selection of patients^17^, which corroborates our approach. Compared to classifying patients according to tumor stage and grade, which is common in adjuvant trials, *MFI2-AS1* is a more potent classifier for ccRCC recurrence risk. Compared to classifications that use multiple genes^14^, *MFI2-AS1* status is easier to determine with common methods such as quantitative PCR, and sill provides high discriminatory power. Using the Leibovich score and *MFI2-AS1* status allows for an unprecedented stratification of patients according to their risk of relapse and should be useful in clinical practice.

Detectable expression of *MFI2-AS1* may result from amplification of its locus at 3q29, an event that may promote oncogenic mechanisms. Notably, we reported that expression of *MFI2-AS1* was associated with expression of acute-phase proteins such as *IL6* and *SAA1*, which might indicate that *MFI2-AS1* is involved in the positive regulation of genes responsible for tumor-promoting inflammation, a poor prognosis factor in ccRCC^35^. In addition, the correlation between *MFI2-AS1* expression and metastatic disease indicate that *MFI2-AS1* might be involved in tumor progression. Recent data from the literature corroborate these findings, with reported association between expression of *MFI2-AS1* and proliferation of osteosarcoma cell lines^26^.

The results of this study are robust as it was conducted in two large independent cohorts with fresh frozen tissue samples. Modification of the endpoints to another reference classification would have likely led to similar outcomes. Indeed, Leibovich and UISS score are based mostly on similar criteria, notable T stage, nodal involvement and pathological grade. In addition, their concordance index are very similar, which indicate comparable discriminatory power^15^. This study did not prospectively recruit the two cohorts. However, the characteristics of the patients are similar to those encountered in the literature^36^. The FU for each patient was not standardized, but patients were monitored according to the standard of care, which included receiving a CT scan every 3–6 months. Samples from ten patients (5.5%) in the validation cohort could not be analyzed for technical reasons (insufficient RNA yield or low quality data), and 27 (13%) patients were excluded due to insufficient follow-up. Our consideration of tumor heterogeneity, an increasing challenge for cancer diagnosis and treatment^29^, showed that detection of *MFI2-AS1* was found recurrently across multiple regions of primary tumors that had documented *MFI2-AS1* expression. The differential quantitative expression of *MFI2-AS1* could be explained by the variation of the amount of tumor cells in each sample, as we did not perform microdissections, but also by intratumor heterogeneity for *MFI2-AS1* expression levels. Dedicated studies will be useful to investigate tumor heterogeneity for *MFI2-AS1* expression, not only in the primary tumor, but also between primary and metastatic sites and at different time points, in order to better understand the role of *MFI2-AS1* during cancer progression.

Further studies will be important to prospectively validate the impact of *MFI2-AS1* stratification in adjuvant clinical trials. Liquid biopsies are under study to detect lncRNA transcripts in circulating blood^37^, which will vastly improve the availability of prognostic tools. Improved comprehension of the non-coding genome will help determine the oncogenic mechanisms associated with *MFI2-AS1* expression, as well as their involvement in tumor immunity. Our work highlights the involvement of the non-coding genome in cancer, an ever-expanding area of research that will help to shape the future of medical oncology.

## Electronic supplementary material

Supplementary information

Long non-coding RNAs associated with prognosis and tumor stage in the training set

Functional annotation of differentially expressed genes according to MFI2-AS1 expression in the training set
