# Supplementary material for: Expression of long non-coding RNA MFI2-AS1 is a strong predictor of recurrence in sporadic localized clear-cell renal cell carcinoma
Source: Sci Rep. 2017 Aug 17;7:8540. doi: 10.1038/s41598-017-08363-6 (PMC5561098; doi:10.1038/s41598-017-08363-6)

Expression of long non-coding RNA *MFI2-AS1* is a strong predictor of recurrence in sporadic localized clear-cell renal cell carcinoma

Ronan Flippot, Roger Mouawad, Jean-Philippe Spano, Morgan Rouprêt, Eva Compérat, Marc-Olivier Bitker, Jérôme Parra, Christophe Vaessen, Frederick Allanic, Quentin Manach, Nizar M. Tannir, David Khayat, Xiaoping Su & Gabriel G. Malouf

Supplementary Tables.

Supplementary Table 1. Intratumor heterogeneity of *MFI2-AS1* expression in the validation cohort.

Sample name	Region of the primary tumor	*MFI2-AS1* expression	Normalized quantitation of *MFI2-AS1* expression
HET11	1	Yes	0,51
	2	Yes	12,73
	3	Yes	10,56
HET1	1	Yes	3,58
	2	Yes	6,32
HET16	1	Yes	9,92
	2	Yes	6,15
HET2	1	Yes	8,22
	2	Yes	0,88
	3	Yes	80,45
	4	Yes	1,10
HET17	1	Yes	1,32
	2	Yes	7,94
	3	No	0,00
HET5T	1	Yes	0,13
	2	Yes	0,60
	3	Yes	0,93

Supplementary Table 2. Hazard ratios for disease-free survival according to Leibovich (< 5 or ≥ 5) and *MFI2-AS1* (expression or no expression) stratification. NS: not significant

Group comparison	Hazard Ratio [95% confidence interval]	p-value
L+M+ vs. L+M-	12,16 [3,76 - 39,34]	<0·0001
L+M+ vs. L-M+	10,82 [2,61 - 44,85]	<0·0001
L+M+ vs. L-M-	15,28 [2,61 - 89,57]	<0·0001
L+M- vs. L-M+	0,56 [0,15 - 2,15]	NS
L+M- vs. L-M-	0,99 [0,22 - 4,40]	NS
L-M+ vs. L-M-	1,70 [0,64 - 4,49]	NS

Supplementary Figures

Supplementary Figures Legends

Supplementary Figure 1. Example of qPCR curves for *MFI2-AS1* assessment in the validation cohort. A) No detectable *MFI2-AS1* expression B) Detectable *MFI2-AS1* expression

Supplementary Figure 2. ROC curves for recurrence prediction according to Leibovich classification and *MFI2-AS1* status in the validation cohort. A) Combined Leibovich (<5/≥ 5) and *MFI2-AS1* (expression/no expression) classification. B) Leibovich classification (<5/≥5) alone. C) *MFI2-AS1* (expression vs. no expression). D) *MFI2-AS1* (continuous).

Supplementary Figure 3. Disease-free survival according to *MFI2-AS1* and distinct Leibovich classification cutoffs in the validation cohort. A) *MFI2-AS1* and Leibovich low, intermediate or high B) *MFI2-AS1* and Leibovich low or intermediate + high

Supplementary Figure 1.

A.

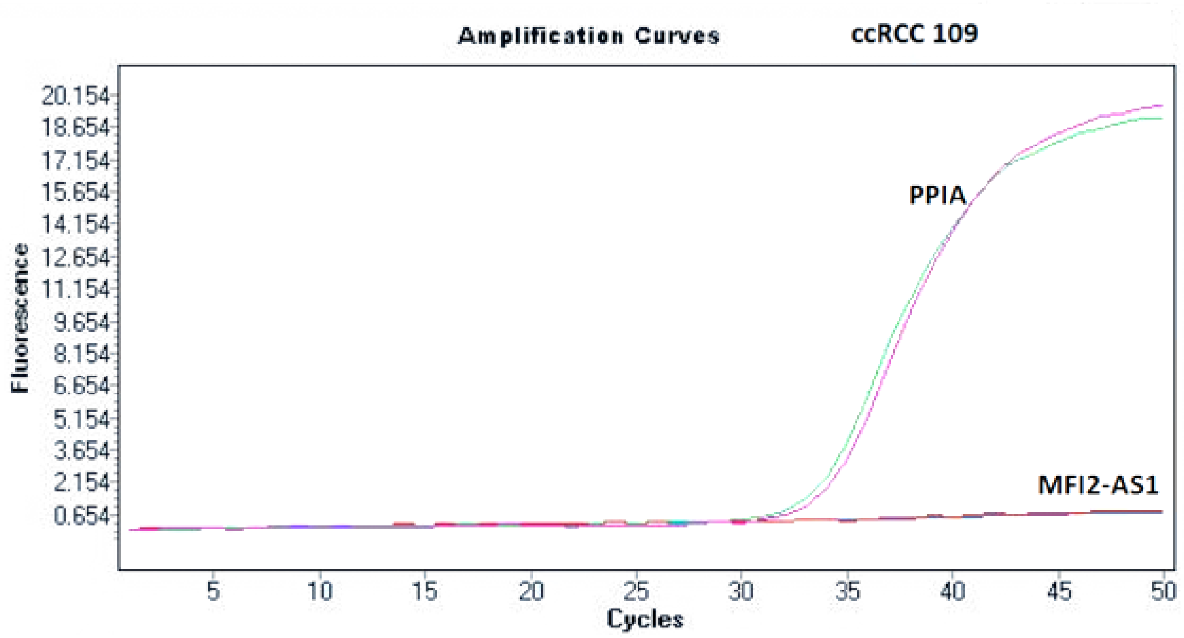

B.

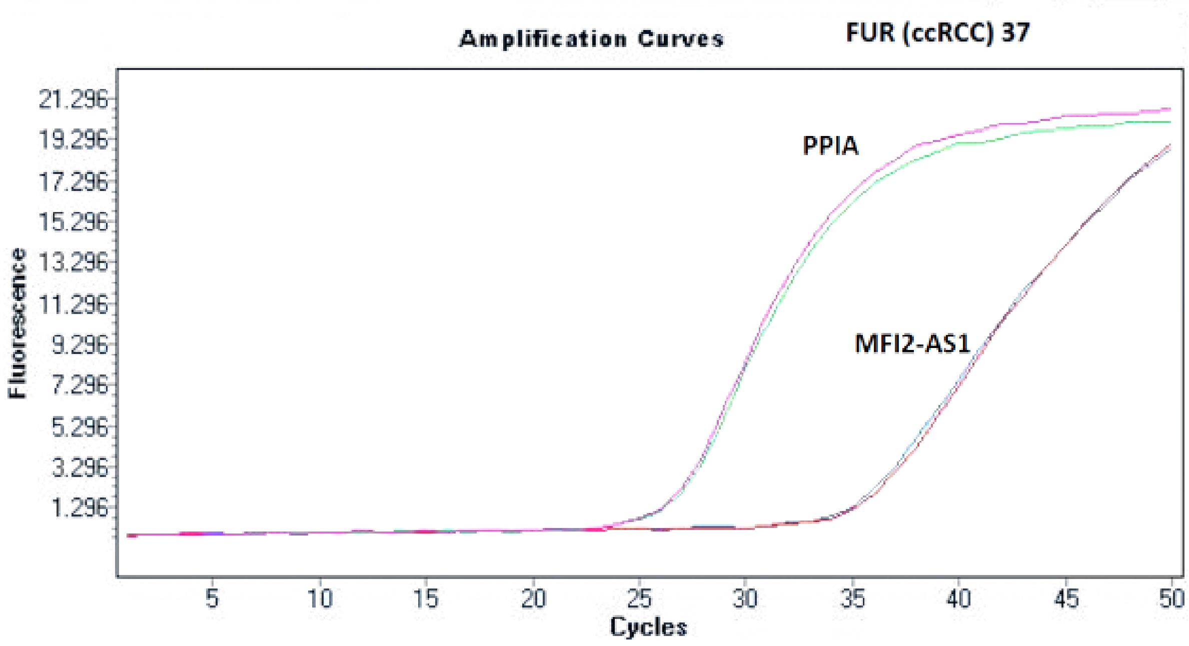

Supplementary Figure 2.

A.

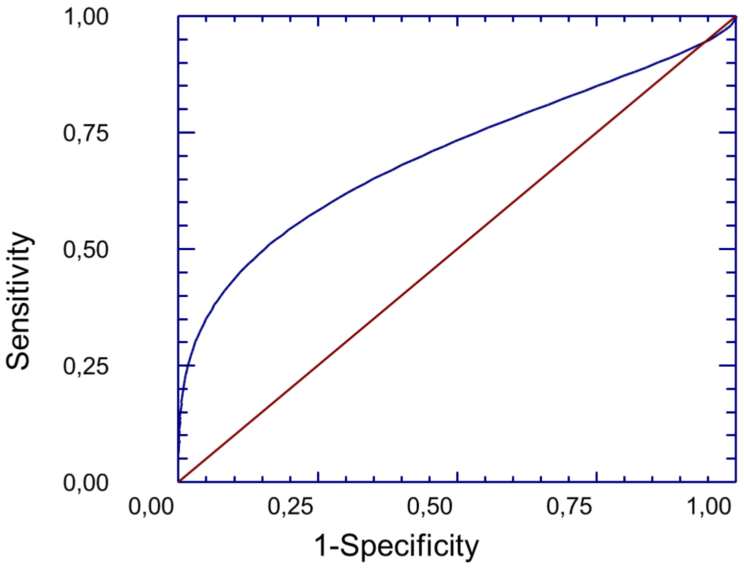

Binormal estimate of AUC 0,701960

B.

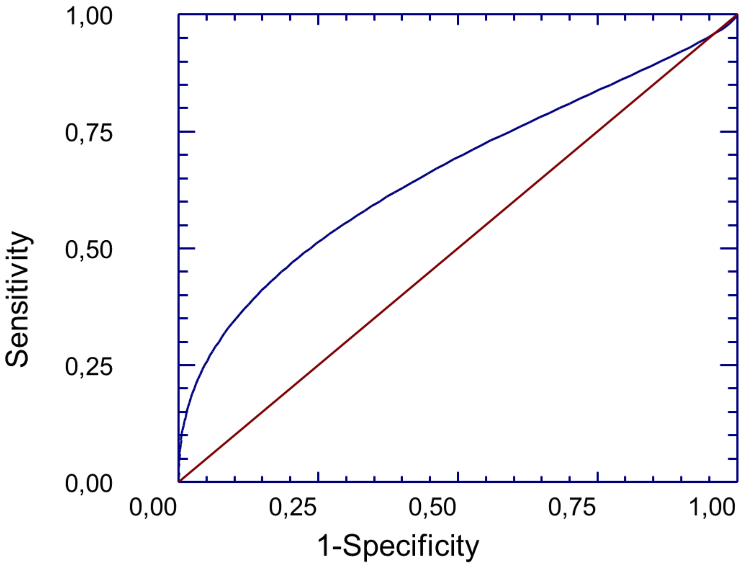

Binormal estimate of AUC 0,66100

C.

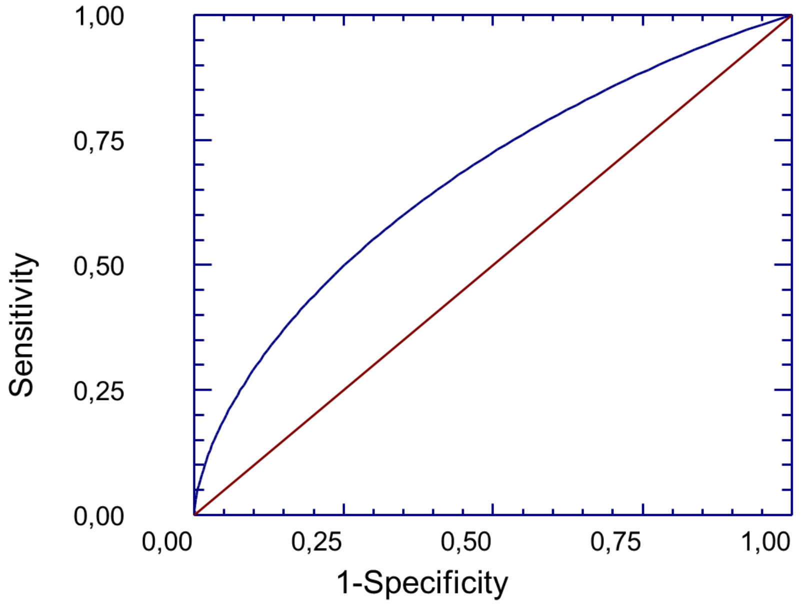

Binormal estimate of AUC 0,67186

D.

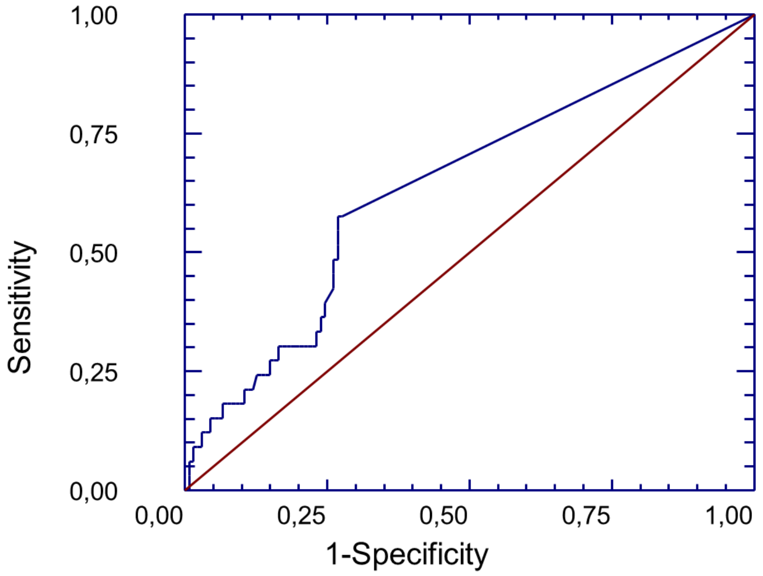

Empirical estimate of AUC 0,63806

Supplementary Figure 3.

A.

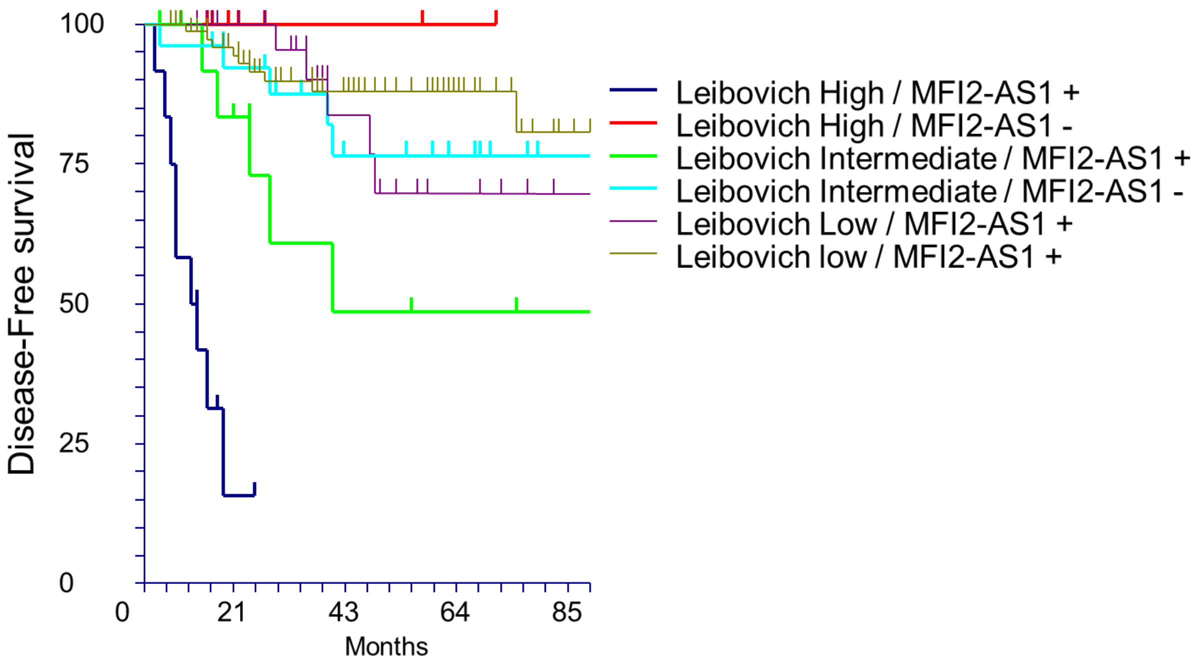

B.

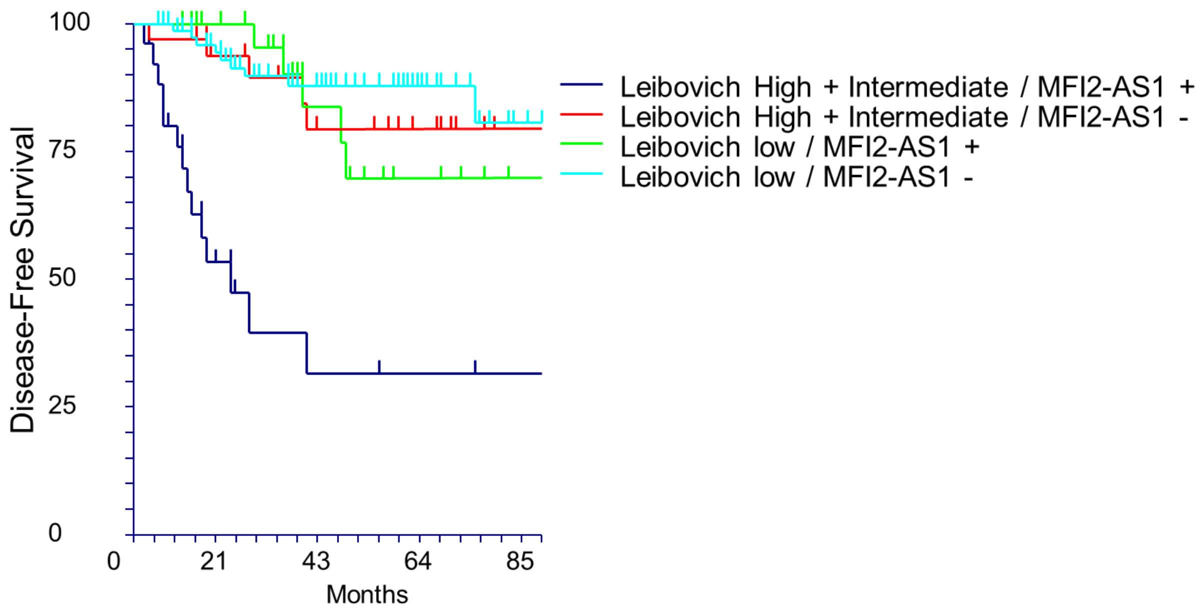

Supplement: Supplementary file 1 — Supplementary information [file 41598_2017_8363_MOESM1_ESM.pdf]
